# Registration of Health-Related Quality of Life in a Cohort of Patients Undergoing Cholecystectomy

**DOI:** 10.5402/2011/507389

**Published:** 2011-06-21

**Authors:** Simon Henry Pålsson, Ib Rasmussen, Patrik Lundström, Johanna Österberg, Gabriel Sandblom

**Affiliations:** ^1^Department of Surgery, Lund University Hospital, 22242 Lund, Sweden; ^2^Department of Surgery, Uppsala University Hospital (Akademiska), 75185 Uppsala, Sweden; ^3^Department of Surgery, Mora Hospital, 79285 Mora, Sweden; ^4^CLINTEC, Karolinska Institutet, 14186 Stockholm, Sweden

## Abstract

*Background*. Assessment of gallstone surgery's impact on quality of life (QoL) requires a reliable instrument with sufficient responsiveness. The instrument should also enable estimation of each individual's expected condition in an unaffected state. *Materials and Methods*. The Swedish Register for Gallstone Surgery and ERCP (GallRiks) registers indications, complications, results, and QoL-outcome of gallstone surgery. In 2008, 68 hospitals were registered in GallRiks. Between 2007 and 2008, SF-36 (a short form health survey) was filled in 1-2 weeks pre- and 6–9 months postoperatively at five of the units. Expected scores were determined from an age- and gender-matched Swedish population (AGMSP). *Results*. Of the 330 patients, 212 responded to SF36 pre- and postoperatively (RR = 64%; 212/330). Standardized response means ranged from 0.20 to 0.93 for the SF-36 subscores. Highest responsiveness was seen for bodily pain. Preoperatively, all subscores were significantly lower than in the AGMSP (all *P* < .05). Six months postoperatively, there was no significant difference between any of the observed and expected quality of life subscales. *Conclusion*. SF-36 is a useful instrument for measuring the impact of gallstone surgery on QoL. The postinterventional health status equalled or even exceeded the AGMSP for all subscales.

## 1. Introduction

Cholecystolithiasis is common in the Swedish population. The prevalence has been estimated to be 53% for women and 32% for men, respectively, at the age of 75 years or more [1]. The clinical manifestation of gallstone disease varies from complete absence of symptoms to daily pain attacks which may seriously affect health-related quality of life (HRQoL) and cause secondary complications with high morbidity and even some mortality. Most gallstones, however, have little or no symptoms [2]. Since gallstone disease may have a negative effect on the quality of life and could develop into a potentially lethal disease, the question is still whether or not this group should be a target for interventional surgical procedures such as cholecystectomy [2]. After the introduction of laparoscopic cholecystectomy with all the benefits of this approach, the threshold for surgery has been lowered and indications widened which makes it even more important to determine, in the preintervention setting, who is most likely to benefit from surgery [3–6]. 

To determine the impact of gallstone surgery on quality of life, a standardized questionnaire with high reliability and validity is required. In medical decision-making, it is important to have an instrument for measuring patient-perceived outcome of treatment, and for assessing each individual's expected condition in an unaffected state to serve as a reference with which the results of medical and surgical procedures may be compared. The aim of this study was to see which aspects of HRQoL are affected by gallstone surgery, to determine whether the responsiveness of SF-36 is sufficient to use as an instrument for measuring the impact of gallstone surgery on HRQoL, and to see if patients who undergo gallstone surgery attain the same HRQoL scores as the general population.

## 2. Materials and Methods

This study is based on data from *The Swedish Register for Gallstone Surgery and ERCP (GallRiks)*, founded in May 2005 and supported by the Swedish Surgical Society. Since its foundation it has received financial support from the Swedish National Board of Health and Welfare. It covers open and laparoscopic surgery of the gallbladder as well as all endoscopic interventions of the bile ducts. GallRiks is an internet application with online registration of procedures and followup as well as electronic reports on demand. The aim of the register is to provide participating hospitals and doctors with continuously updated results regarding indications for surgery, surgical, and endoscopic methods, complications and patient satisfaction with the care provided. The results are presented and compared to national data over time and can thereby be used to support efforts for quality improvement at each hospital. 

Another aim is to use the national database for scientific analysis of the epidemiology and treatment of gallstone disease in Sweden. 

A programme for continuous validation of data has been installed. Four experienced doctors make hospital visits to ensure that adequate resources have been assigned for registration and followup, and to compare a sample of 25 medical records with entries in the database. Each hospital is visited once every 3 years. Results from the first 20 hospitals visited indicate complete match between the medical records and the database in 98%. 

By the end of 2008, 68 hospitals were registered in GallRiks. This study is based on data assembled from GallRiks 2006–2008. The number of cholecystectomies reported (acute and elective) was 28176 (86% of implemented operations, all procedures performed in Sweden 2008). Registered data included age, gender, medical history, indication for surgery, operation method and pre- and postoperative complications within 30 days after surgery. Indications for surgery were biliary colics without secondary complications, cholecystitis and/or pancreatitis and/or jaundice, acalculous cholecystitis, and gallbladder polyp or suspected tumour. 88% of the interventions were performed by nonconverted laparoscopy (Table 2).

Since 2006 the registration has been extended with the option to register health-related quality of life prior to surgery and, patients from 16 of the hospitals included in GallRiks were requested to reply to the SF-36 questionnaire 1-2 weeks preoperatively and 6–9 months after surgery. The questionnaire were sent by mail or distributed at the hospital. The registration of quality of life is not yet nationwide but limited to a number of units where registration is performed as a pilot study.

The SF-36 is a generic multipurpose, short-form health survey with 36 items, used to register QoL. It yields an 8-scale profile of functional health and well-being scores as well as psychometrically based physical and mental health summary measures. SF-36 has proven useful in surveys of general and specific populations, comparing the relative burden of diseases and in differentiating the health benefits produced by different treatments. Three scales of SF-36—physical functioning (PF), role-physical (RP), and bodily pain (BP)—correlate mostly with the physical component summary (PCS) measure, and the mental component score (MCS) correlates mostly with mental health (MH), role-emotional (RE), and social functioning (SF). Three of the scales—vitality (VT), general health (GH), and SF—have correlations with both components. The theoretical maximum score is 100 for each subscale [7]. SF-36 was also a recommended validated QoL-instrument for gallbladder disease by the European Association for Endoscopic Surgery (EAES) [8]. A standard Swedish population answered the SF-36 in a previous study [9], and it was therefore possible to compare our patient group with a standard population of similar age and gender. The patients who filled in the questionnaire before surgery represent a nonselected population. 

From GallRiks database five hospitals with the highest SF-36 response frequency (Halmstad, Jönköping, Mora, SU/östra Gothenburg and Söder Stockholm) were selected for this study. 

## 3. Results

Altogether 817 patients were operated upon during 2008 at the units covered by the study. 330 patients responded to the questionnaire preoperatively. Of the preoperative responders, 212 responded postoperatively, yielding a postoperative response rate of 64% (Table 1). All data presented here are based on those who responded pre- and postoperatively.

Mean age was 51 years, standard deviation 16 years. There were 80 men and 250 women. Indication for surgery and method of approach are presented in Table 2. Postoperative complications were registered following 20 (7.3%) procedures. 

**Figure 1:**
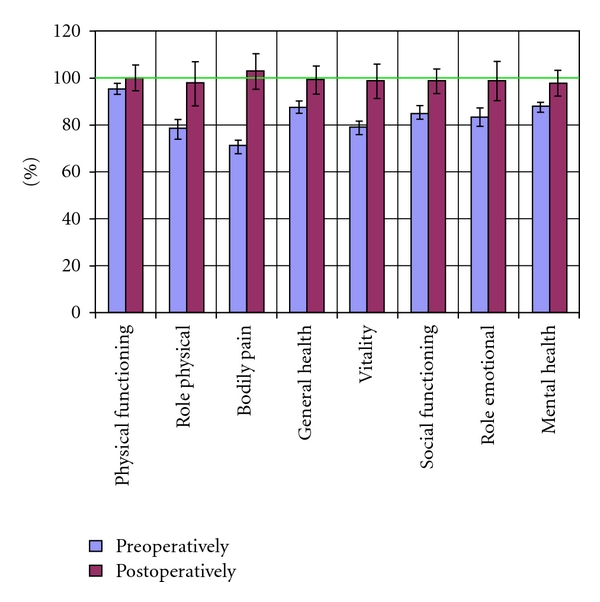
Mean subscale values ±95% confidence interval. Values adjusted for expected values derived from the general age- and gender-matched population [9].

Each patient completed the SF-36 questionnaire before surgery and 6 months postoperatively. Significant postoperative improvement in quality of life was seen in all SF36 domains (*P* < .05) (Figure 1). Most pronounced was the improvement in bodily pain (0.93; Table 3). The value 100 means that the mean postoperative increase is equal to the standard deviation for each subscale thus in this study equals the standard Swedish population in a healthy state. The higher the score the more impact from the intervention. Standardized response means show the responsiveness of a certain interventions' effect on HRQoL. High responsiveness corresponds to a great interventional effect and proves that the instrument used for measuring the improvement after gallstone surgery, in this case SF-36, is reliable.

## 4. Discussion

Gallstone surgery, as performed in this cohort, led to a significant improvement in all 8 SF-36 domains. The postinterventional health status equalled or even exceeded the age- and gender-matched general population in all subscales. Gallstone surgery, as performed in this unselected population, thus restored the perceived HRQoL to a level equal to or even higher than the normal population, QED. The greatest improvement and also the highest responsiveness were seen for bodily pain. The most sensitive indicator when assessing the effect of gallstone surgery is therefore bodily pain. Whether or not a placebo effect contributed to this improvement in HRQoL is impossible to say. Nevertheless, each patient's subjective improvement in HRQoL is measurable with the SF-36. Gallstone-related bodily pain is the most sensitive indicator of improvement after gallstone surgery and should therefore be included, together with other symptoms, when considering indication for surgery. Improvement in HRQoL in other subscales is less likely to be caused by the surgical intervention. 

There are also other instruments that have been suggested as standardized instruments for evaluating the outcome after gallstone surgery, in particular Gastrointestinal Quality of Life Index (GIQLI). Even if GIQLI is more disease-specific than SF-36, SF-36 has the advantage of being accessible in more languages and more widely established than GIQLI. 

We know that gallstone disease may have a profoundly negative impact on the quality of life, especially in symptomatic patients with a history of biliary colic and/or complications of the disease [11], and that patients with symptomatic cholecystolithiasis and low surgical risk have the largest benefit from gallstone surgery [3, 12]. Bitzer et al. showed that some patients have very high expectations of gallstone surgery, and that 85.7% expect complete relief of impairment symptoms as determined by a lower GSCL score 6 months postoperatively [13]. There are also age- and gender-related differences. Women have a worse preintervention health status than men as measured with SF-36 and GIQLI [14]. Furthermore, women showed less postoperative improvement than men. Older patients also have poorer HRQoL scores and less postintervention improvement [14]. It is difficult to say whether the effect of surgery on HRQoL depends on preoperative selection methods, or that the group of symptomatic carriers contains more individuals with a lower baseline health status than the age- and gender-matched general population.

It may also be that patients selected for gallstone surgery constitute a subgroup of the general population with a high prevalence of abdominal symptoms due to other causes that may be present alongside or even predominate over symptoms directly related to their gallstones. Since gallstones as well as nonspecific abdominal symptoms (e.g., irritable bowel syndrome (IBS), dyspepsia, and motility disturbances) are quite common, the risk of having asymptomatic gallstones and abdominal symptoms of other aetiology concurrently is relatively high. Surgical treatment of gallstones in such a case will not relieve the symptoms, and give a lower posttreatment quality of life score than the general population. On the other hand, if the baseline health status of patients with gallstones is equal to the age- and gender-matched population, there should be no difference between operated patients and the general population. A study performed by Mentes et al. suggested that laparoscopic surgery should also be performed on asymptomatic patients since this group improved in their GIQLI score. The general consensus amongst most authors, however, seems to be that this group should not be treated surgically [11, 15]. Several studies have been performed comparing quality of life before and after a surgical procedure. However, it may be difficult to determine if the improvement in HRQoL seen after surgery is a result of the surgical intervention per se, or if it is mainly a placebo effect? 

There is concern about the risk of inappropriately selecting patients with functional gastrointestinal symptoms for cholecystectomy [1]. The attribution of symptoms related to IBS that frequently coexists with gallstones, to gallstone disease increases the risk of performing cholecystectomy on wrong indications. This has been suspected to be a major cause of the increasing rate of cholecystectomies performed in recent years [16]. A lower threshold for performing surgery after the introduction of the laparoscopic technique may also have influenced the number of cholecystectomies performed [17–19]. A shift in indications for performing cholecystectomy should lead to an increased prevalence of persisting symptoms postoperatively and a discrepancy between the perceived health-related quality of life of those who have undergone cholecystectomy and an age- and gender-matched general population. Such a discrepancy, however, was not seen in our study, at least not to the extent that reduced health-related quality of life, as measured by SF-36, could be seen. Although definite conclusions cannot be drawn without complete coverage, it seems that gallstone surgery performed in the community at large, as in the population studied, is based on indications that are relatively effective in identifying those who are likely to benefit from surgery. However, longer followup is required before definite conclusions can be drawn.

The improvements seen in our study may, to some extent, reflect the regression to the mean phenomenon. Most symptoms tend to vary with time, regardless of treatment or intervention. Since patients contact the health care provider during periods of most intensive symptoms, the timing of the intervention tends to coincide with the period when the natural course of the disease may lead to improvement after a temporary peak giving the impression of a beneficial effect of treatment. The case-mix with acute and chronic gallstone diseases as indications for surgery may also have affected the outcome. It is likely to believe that patients operated for an acute gallstone disease experience a more pronounced improvement in HRQoL than those undergoing an intervention for a more relative indication. The placebo effect should also be considered as a partial explanation for the improvement seen. 

A recent similar study on responsiveness after cholecystectomy from Taiwan [20] found standard response means close to those in our study. The relatively higher standardized response mean for bodily pain was also confirmed, with values ranging from 0.23 to 0.93. However, as the standardized response means were slightly lower than those seen for Gastrointestinal Quality of Life Index (GIQLI), the Taiwanese study concluded that disease-specific measures should be weighted more heavily than generic measures.

The population used as background to assess the expected posttreatment quality of life level is very close to the study population in time and place. The study cohort was assembled from rural as well as urban communities. Nevertheless, although the response rate was relatively high, the health-related quality of life of the nonresponders may have differed from the responders. From a theoretical point of view it may be possible that the nonresponders represent a healthier subgroup with a higher HRQoL than the standard population in an unaffected state or the opposite way. The routines for mail correspondence may also have differed between the units. No national guidelines were drawn at the time for the study concerning routines for reminding letters. 

## 5. Conclusion

Our study shows that SF-36 is useful for evaluating the impact of gallstone surgery on HRQoL. All aspects covered by SF-36 are improved postoperatively, in particular bodily pain. The HRQoL as perceived by a community-based population after having undergone gallstone surgery equalled or even exceeded the age- and gender-matched general population for all subscales.

## Figures and Tables

**Table 1:** Response rates at the different units.

Hospital	Preoperative registrations	Complete registrations	Response frequency
Halmstad	*N* = 72	*N* = 51	(51/72) = 71%
Jönköping	*N* = 72	*N* = 53	(53/72) = 74%
Mora	*N* = 109	*N* = 68	(68/109) = 62%
Södersjukhuset	*N* = 23	*N* = 12	(12/23) = 52%
Sahlgrenska/Östra	*N* = 54	*N* = 28	(28/54) = 52%
TOTAL	*N* = 330	*N* = 212	(212/330) = 64%

**Table 2:** Baseline data. Approaches and indications for cholecystectomies performed. It starts with mostcommon approach/indication.

	*N*	%
Indication		
Attacks of biliary colic without secondary complication	262	79.4
Cholecystitis and/or pancreatitis and/or jaundice	59	17.9
Acalculous cholecystitis	1	0.3
Gallbladder polyp or suspected tumor	3	0.9
Other/unclear	5	1.5
Total	330	100

Approach		
Laparoscopic	290	87.9
Laparoscopic, converted to open	28	8.5
Conventional open	9	2.7
Minilaparotomy (Incision < 8 cm)	1	0.3
Not registered	2	0.6
Total	330	100

**Table 3:** Standardized response means (SRMs). The higher the score the more impact from the intervention. Standardized response means show the responsiveness of a certain parameter's effect on HRQoL. Thus equals the estimated HRQoL for the standard Swedish population. Responsiveness is showing the validity of SF-36 on gallstone surgery. High responsiveness proves that the instrument used for measuring the improvement after gallstone surgery, in this case SF-36, is reliable.

Subscale	Standardized response mean	95% Confidence interval
Physical functioning	0.22	0.11–0.33
Role physical	0.49	0.37–0.61
Bodily pain	0.93	0.78–1.07
General Health	0.21	0.10–0.32
Vitality	0.38	0.26–0.50
Social Functioning	0.46	0.33–0.59
Role-emotional	0.31	0.18–0.45
Mental health	0.26	0.15–0.36
Physical component score	0.53	0.41–0.65
Mental component score	0.29	0.17–0.41

Evaluation of SRM [10]:

(i) <0.20: trivial effect;

(ii) 0.20–0.50: small effect;

(iii) 0.50–0.80: moderate effect;

(iv) >0.80: large effect.
